# 928. Clinical Characteristics and Microbiology Testing Patterns Among Transplant Recipients Admitted to Acute Care Hospitals for Suspected Infection

**DOI:** 10.1093/ofid/ofab466.1123

**Published:** 2021-12-04

**Authors:** T Matthew Hill, Erick R Scott, Sivan Bercovici

**Affiliations:** 1 Karius, Inc, Austin, Texas; 2 Karius, Redwood City, California

## Abstract

**Background:**

Solid organ transplant (SOT) is a growing option for patients with end-stage organ diseases. Immunosuppressive therapy (IT) is utilized in this population to minimize risk of allograft rejection, which increases infection risk particularly of atypical pathogens that can complicate the infection-related diagnostic journey. The purpose of this analysis was to evaluate baseline clinical characteristics and microbiological testing utilization patterns among a cohort of patients with a history of SOT and IT.

**Methods:**

This retrospective cohort study utilized a US hospital-based, service-level database. Patients were selected from a subsample of database facilities utilizing plasma microbial cell-free DNA diagnostic assays. The study period was 1/1/2017-3/21/2020. Eligible patients were identified by 1^st^ observation of SOT status and IT. Subsequent inpatient admissions for suspected infection were analyzed.

**Results:**

We identified 749 patients with SOT history and use of IT, 56.4% were male, and the mean age was 52.8 (18.7) years. Kidney was the most prevalent transplant category (49.1%), followed by liver (14.1%), lung (10.9%), and heart (10.3%), and 9.7% were multi-organ. Patients experiencing multiple transplants had the most chronic conditions with a mean Elixhauser comorbidity score of 26.3 (14.7). The median length of stay was 4 [3-7] days. The median number of tests per encounter was 6 [IQR=3-11]. Culture was the most utilized test category (2 [1-4]). Blood culture was the highest utilized culture and overall test at 13.5% of all tests observed, while CMV PCR (7.8%) and multi-panel EIA (2.7%) were the most frequent molecular and antigen tests, respectively. Lung transplant recipients had the greatest utilization of tests overall (9 [3.5-17]) versus other transplant categories (6 [3-10]), consistent with the observed test rate in the 1^st^ 48 hours of presentation (4 [1-7] vs. 2 [1-5]).

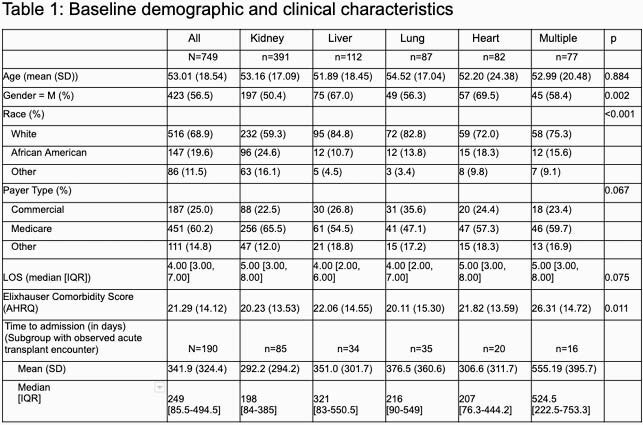

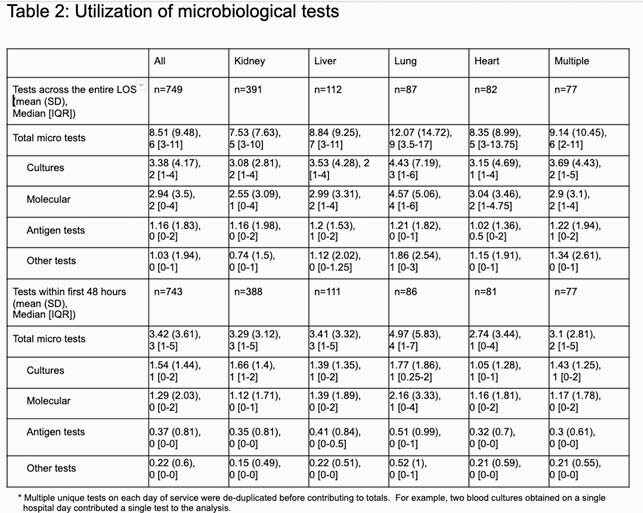

**Conclusion:**

This analysis suggests that the infection-related diagnostic journey among patients with a history of SOT involves high utilization of microbiological testing, with greater utilization among lung transplant recipients versus other SOT recipients. Variation in clinical characteristics and microbiological testing patterns were observed across SOT categories.

**Disclosures:**

**T Matthew Hill, PharmD, PhD**, **Karius, Inc** (Employee, Shareholder) **Erick R. Scott, MD, MHS**, **Karius, Inc** (Employee, Shareholder) **Sivan Bercovici, PhD**, **Karius** (Employee)

